# Effect of diversity and missing data on genetic assignment with RAD-Seq markers

**DOI:** 10.1186/1756-0500-7-841

**Published:** 2014-11-25

**Authors:** Balaji Chattopadhyay, Kritika M Garg, Uma Ramakrishnan

**Affiliations:** Ecology and Evolution, National Centre for Biological Sciences, TIFR, Bellary Road, Bangalore, 560065 India

**Keywords:** RAD-Seq, Missing data, Genetic assignment, *Cynopterus*

## Abstract

Reduced representation libraries are being used as a preferred source of markers to address population genetic questions. However, libraries of RAD-Seq variants often suffer from significant percentage of missing data. In addition, algorithms used to mine SNPs from the raw data may also underscore biological variation. We investigate the effect of biological diversity in mining SNPs from the program STACKS and the effect of missing data on individual assignment implemented in STRUCTURE. We observed that changing diversity parameters in STACKS significantly alters the number of SNPs discovered and allowing for higher percentage of missing data retrieves more loci and possibly more power for individual assignment.

**Electronic supplementary material:**

The online version of this article (doi:10.1186/1756-0500-7-841) contains supplementary material, which is available to authorized users.

## Correspondence

### RAD-Seq and missing data

Reduced representation genomic libraries are increasingly used to answer diverse questions in evolutionary biology, which remained unresolved otherwise. Various restriction-site based genome scans have become standard tools for both population genetic and phylogenetic analyses [1]. Although extremely useful, these techniques are cost prohibitive and generation of data from hundreds of individuals across multiple runs may not really be an option for many research labs. Besides, technical issues related to data generation result in the requirement of multiple runs to troubleshoot, thereby increasing the cost manifold.

RAD-Seq (restriction site-based reduced representation genomic libraries) generates tens of thousands of loci per individual, but overlapping loci across all individuals are much fewer, resulting in significant missing data. Since missing data could impact inference, it is important to test its effect on the analysis. While missing data may not significantly impact phylogenetic inference [2], other forms of population genetic inferences remain untested.

We compared genetic assignment and group membership of individuals as inferred by nuclear autosomal microsatellite loci and genome-wide single nucleotide polymorphisms (SNPs) sites obtained from RAD-Seq for ten individuals of two Cynopterine fruit bat species *Cynopterus sphinx* and *C. brachyotis* (Additional file 1: Table S1). We assessed the effect of missing data on group assignment in RAD-Seq. Because software tools are used to ascertain SNPs in RAD data, SNP calling is impacted by assumptions of the software tools. Thus the number and quality of markers obtained is highly influenced by the software. We also investigated how such assumptions impact results, specifically the effect of change in diversity parameters (mismatch between loci) within a widely used tool for mining SNPs with RAD-Seq data, STACKS 1.09 [3].

### Library preparation and analyses

As an ongoing project to estimate gene flow and understand its dynamics between these two bat species in sympatry, we used data from ten individuals from a pool of 387 genotyped individuals (Additional file 1). We prepared standard RAD libraries with individual barcodes (Additional file 1: Table S1). We performed a standard paired end run (single lane) in HiSeq 1000. Data output was 91 million reads, but paired-end quality was poor and was not considered in further analyses (Additional file 1). We subsequently analyzed ~47 million forward reads. We observed more than 90% data loss due to ambiguous barcode (Additional file 1). The average number of reads per individual was 468,612.3 (range: 366,389 to 731,138, Additional file 1: Table S1). Within the STACKS pipeline, the basic algorithm for arranging reads into stacks depends upon absolute nucleotide matches and has often been regarded as conservative [4]. Additionally, there is evidence that when sequence diversity is high (as in our case where we are examining sequences from two distinctly diverged genomes) stacks may remove a majority of the loci from its analysis, or may separate single locus into two [5].

We assessed the sensitivity of STACKS to these deviations in differentiating between these well-defined taxa. First we obtained SNPs from different run parameters, with 50% missing data. Further, for the parameter combination that provided meaningful number of loci, we obtained SNPs with different extent of missing data (10%, 30%, 50%, 70% and 90%). For all these parameter sets we performed independent STRUCTURE runs (50,000 burnin and 100,000 mcmc) considering two genotypic clusters (K =2) [6]. Each run was replicated five times and the mean of ancestry coefficient across all these replicates was used to obtain trends.

### Results

**Figure 1:**
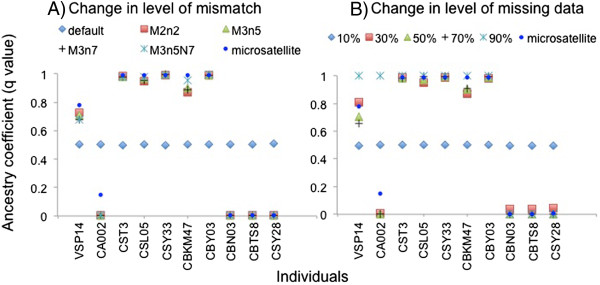
**Ancestry coefficient for individuals (obtained from structure) A) with increase in number of mismatches allowed to generate loci in Stacks (default: two mismatches between reads within a locus, M2n2: two mismatches between reads within a locus and two mismatches between loci when comparing across individuals, M3n5: three mismatches between reads within a locus and five mismatches between loci when comparing across individuals, M3n7: three mismatches between reads within a locus and seven mismatches between loci when comparing across individuals and M3n5N7: three mismatches between reads within a locus and five mismatches between loci when comparing across individuals and additionally allowing seven mismatches to align secondary reads to generate a locus) and B) increasing the proportion of missing data.**

We obtained 17 to 2954 loci with all parameter combinations tested in our study (Additional file 1: Table S2) with substantial variation between individuals. Our results suggest that in the absence of any mismatch between loci (default settings in denovomap.pl) the SNPs obtained cannot differentiate between both species. However, species differentiation is accurate when mismatches are allowed and there is also no significant effect of varying degrees of mismatches (Figure 1A). We observe that beyond the mismatch parameter of M = 3 (mismatches between reads within a locus) and n =5 (mismatches between loci when comparing across individuals) the number of SNPs (or loci) do not increase significantly (Additional file 1: Table S2 and S3). Presence of mild to considerable extent of missing data (Additional file 1: Table S4) also did not adversely affect the assignment scores in Structure and most individuals were ascribed group memberships in agreement with the microsatellite dataset. However one individual (CA002) considered as genetic intermediate (microsatellite data) was consistently assigned to *C. brachyotis* based on the SNP analysis (Figure 1, Additional file 2: Figure S1). Further, we also observed that allowing for 10% missing data returned too few loci (228 loci) to differentiate between two species, whereas 90% missing data obliterated any intermediate ancestry coefficients (Figure 1B). It appears that too much missing data reduces the power of assignment to a considerable extent. However, the cutoff percentage of missing data from STACKS does not represent the actual extent of missing data (Additional file 1: Table S4). Though the cutoffs vary greatly in stringency (10% to 90%) the actual extent of missing data do not vary drastically between the datasets (55% to 74%). This may be due to the fact that in our analysis a locus in STACKS is recognized if it a present in at least one of the species. Thus the percentage cutoff only corresponds to the species in which the locus is present and not the complete dataset. As both populations are not of the same sample size the extent of missing data is greater than indicated by the cutoff. Rather, the cutoffs represent an increase in number of loci when the cutoff parameters become less stringent. We conclude that biological diversity should be taken into consideration while generating SNPs in STACKS (similar checks should be performed with other SNP calling programs as well) and generally RAD-Seq population genomic datasets with considerable missing data may actually mine considerably higher number of loci and have more power [7] to perform population genetic analysis like assignment tests.

### Ethics statement

The sampling were approved by the institutional ethics committees (Internal Research Review Board (IRB), Ethical Clearance (EC), Biosafety and Animal Welfare committee approval to BC dated 21-11-2005 Madurai Kamaraj University and Institutional Animal Ethics Committee (IACE) to UR id UR-3/2009, National Centre for Biological Sciences).

### Data accessibility

Raw sequence reads have been deposited in the Sequence Read Archive (SRA) (accession no. SRP042963).

## Electronic supplementary material

Additional file 1:**Supplementary information. Table S1.** Details of samples used for RAD-Seq library preparation. **Table S2.** Number of locus per samples for each data set. Table S3: Number of SNPs obtained in stacks by varying different parameters in denovomap.pl program in STACKS. Table S4: Number of SNPs obtained in stacks by varying the level of missing data. The average level of missing data was calculated in PLINK 1.07 [7] (url: http://pngu.mgh.harvard.edu/purcell/plink/). (DOCX 115 KB)

Additional file 2: Figure S1: Bar plot of ancestry coefficient for individuals a) with increase in number of mismatches allowed to generate loci in Stacks and b) increase in proportion of missing data. (TIFF 554 KB)
