# Supplementary material for: Effect of diversity and missing data on genetic assignment with RAD-Seq markers
Source: BMC Res Notes. 2014 Nov 25;7:841. doi: 10.1186/1756-0500-7-841 (PMC4256836; doi:10.1186/1756-0500-7-841)
Supplement: Supplementary file 1 — Additional file 1:Supplementary information. Table S1. Details of samples used for RAD-Seq library preparation. Table S2. Number of locus per samples for each data set. Table S3: Number of SNPs obtained in stacks by varying different parameters in denovomap.pl program in STACKS. Table S4: Number of SNPs obtained in stacks by varying the level of missing data. The average level of missing data was calculated in PLINK 1.07 [7] (url: http://pngu.mgh.harvard.edu/purcell/plink/). (DOCX 115 KB) [file 13104_2014_3365_MOESM1_ESM.docx]

**METHODS**

*Sampling:*

We collected tissue samples from bats of both species across their range in India. We sampled and genotyped 387 individuals of which 10 individuals were used in the present study.

*DNA extraction and genotyping*

We extracted total genomic DNAusing the Qiagen tissue extraction kit (QIAmp DNA) following manufacturer’s protocol. We amplified three tri- and six tetra- nucleotide repeat loci, previously developed for *C. sphinx* [1], either using Ampli-Taq Gold DNA polymerase (Applied Biosystems) following Chattopadhyay et al. [2] or PCR Master mix (MM, Qiagen). We genotyped all samples using the ABI3100 XL platform and scored allele sizes using Genemapper v 4.0 (Applied Biosystems). We normalized post genotyping allele sizes using TANDEM [3], which uses a power function to transform allele sizes to integers, while minimizing the rounding errors. We used the normalized allele sizes for subsequent analyses.

*Genetic assignment:*

We used a model-based clustering approach implemented in STRUCTURE 2.3.4 [4] to address the genetic distinctiveness of each species and to further quantify the extent of admixture. We first identified the number of genotypic clusters (K) present within the entire dataset consisting of both pure individuals and intermediates of the two species. We used the second order rate of change of the log probabilities of the data (delta K, [5]) to statistically identify the most likely number of clusters. Further, for each K we obtained and evaluated individual ancestry coefficients (q values) to assign individuals into population clusters. Based on available literature we considered individuals with q values > 0.9 and <0.1 as purebreds and others as possible intermediates.

*Samples used:*

We prepared RAD-seq library for 10 samples, which includes purebred of two species of fruit bats and possible intermediates based on microsatellite based genetic assignment. Details of the samples are given table S1.

*RAD-seq library preparation:*

We followed Etter et al. [6] for RAD library preparation. We used high fidelity eight base pair cutter (SbfI) for restriction digestion. We used six base pair barcode to differentiate between individuals. The barcodes differ by at least two bases (Table S1). We used 200ng of DNA per sample and 75 nM of P1 adapters for library preparation. We carried out eight 30 s on-and-off sonication cycles. We performed 14 cycles for the final PCR amplification. To test the integrity of the library, 4 μl of the final library was cloned using zero blunt end cloning kit (Invitrogen). We sequence 35 positive clones and could obtain nine out of ten barcodes. We performed blastn for the cloned products and observed that majority of the clones contained Chitopteran fragment with intact restriction site, barcodes and sequencing primers. We further performed a quality check using Agilant bioanalyser and observed that our library was of very low template concentration (mean product size 429bp and 2nM). The library was sequenced on an Illumina HiSeq 1000 platform at cCAMP (Bangalore, India).

**REFERENCES**

1. Storz JF: **Variation at tri-and tetranucleotide repeat microsatellite loci in**

**the fruit bat genus *Cynopterus* (Chiroptera: Pteropodidae).** *Molecular Ecology*

2000, **9:**2198-2201.

2. Chattopadhyay B, Garg KM, Doss PS, Ramakrishnan U, Kandula S:

**Molecular genetic perspective of group-living in a polygynous fruit bat,**

***Cynopterus sphinx*.** *Mammalian Biology* 2011, **76:**290-294.

3. Matschiner M, Salzburger W: **TANDEM: integrating automated allele**

**binning into genetics and genomics workflows.** *Bioinformatics* 2009, **25:**1982

1983.

4. Pritchard JK, Stephens M, Donnelly P: **Inference of population structure**

**using multilocus genotype data.** *Genetics* 2000, **155:**945-959.

5. Evanno G, Regnaut S, Goudet J: **Detecting the number of clusters of**

**individuals using the software STRUCTURE: a simulation study.** *Molecular*

*Ecology* 2005, **14:**2611-2620.

6. Etter PD, Bassham S, Hohenlohe PA, Johnson EA, Cresko WA: **SNP**

**discovery and genotyping for evolutionary genetics using RAD sequencing.** In

*Molecular methods for evolutionary genetics.* Springer; 2011: 157-178.

7. Purcell S, Neale B, Todd-Brown K, Thomas L, Ferreira MAR, Bender D, Maller J, Sklar P, De Bakker PIW, Daly MJ: **PLINK: a tool set for whole-genome association and population-based linkage analyses**. *The American Journal of Human Genetics* 2007, **81**(3):559-575.

**TABLES**

| Sample | Species ID | Location | Microsatellite based ancestry coefficient | Barcode for RAD-Seq | Number of reads |
| --- | --- | --- | --- | --- | --- |
| VSP14 | *C. sphinx* | Vishakapatanam | 0.78 | ACACCT | 367,820 |
| CA002 | *C. sphinx* | Agartala | 0.15 | ACAGGA | 371,492 |
| CST3 | *C. sphinx* | Tirunelveli | 0.99 | ACCAGT | 455,699 |
| CSL05 | *C. sphinx* | Lonawala | 0.99 | ACGCTA | 433,887 |
| CSY33 | *C. sphinx* | Yercaud | 0.99 | AGACTG | 439,632 |
| CBKM47 | *C. sphinx* | KMTR | 0.99 | AGCATA | 419,609 |
| CBY03 | *C. sphinx* | Yercaud | 0.99 | AGCTCC | 366.39 |
| CBN03 | *C. brachyotis* | Nilgiris | 0.004 | ACTACC | 543,643 |
| CBTS8 | *C. brachyotis* | Topslip | 0.004 | ACTGAT | 556,814 |
| CSY28 | *C. brachyotis* | Yercaud | 0.006 | AGATAT | 731,138 |

Table S1: Details of samples used for RAD-Seq library preparation.

| Sample | At 50% missing data | | | | | For M3n5 dataset | | | |
| --- | --- | --- | --- | --- | --- | --- | --- | --- | --- |
|  | Default | M2n2 | M3n5 | M3n7 | M3n5N7 | 10% missing | 30% missing | 70% missing | 90% missing |
| VSP14 | 113 | 187 | 197 | 203 | 202 | 19 | 103 | 362 | 838 |
| CA002 | 91 | 189 | 194 | 197 | 198 | 19 | 101 | 394 | 995 |
| CST3 | 133 | 236 | 241 | 246 | 246 | 19 | 123 | 534 | 1380 |
| CSL05 | 122 | 201 | 214 | 214 | 205 | 19 | 109 | 463 | 1221 |
| CSY33 | 132 | 224 | 237 | 240 | 240 | 17 | 118 | 475 | 1263 |
| CBKM47 | 126 | 198 | 207 | 210 | 207 | 18 | 110 | 431 | 1094 |
| CBY03 | 119 | 192 | 197 | 201 | 197 | 19 | 109 | 394 | 826 |
| CBN03 | 440 | 670 | 676 | 673 | 677 | 210 | 227 | 1007 | 1691 |
| CBTS8 | 466 | 694 | 707 | 708 | 716 | 212 | 231 | 1023 | 1723 |
| CSY28 | 557 | 862 | 875 | 872 | 883 | 215 | 233 | 1593 | 2954 |

Table S2: Number of locus per samples for each data set

| Stack depth (m) | Number of mismatch within a locus (M) | Number of mismatch between loci across individuals (n) | Mismatches for secondary reads (N) | Number of SNPs |
| --- | --- | --- | --- | --- |
| 10 | 2 | 0 | 4 | 761 |
| 10 | 2 | 2 | 4 | 1144 |
| 10 | 3 | 5 | 5 | 1169 |
| 10 | 3 | 7 | 5 | 1172 |
| 10 | 3 | 5 | 7 | 1183 |

Table S3: Number of SNPs obtained in stacks by varying different parameters in denovomap.pl program in STACKS.

| % of missing data | Mean level of missing data (in %) | Number of SNPs |
| --- | --- | --- |
| 10% | 66.36 | 228 |
| 30% | 55.37 | 328 |
| 50% | 67.96 | 1169 |
| 70% | 72.71 | 2446 |
| 90% | 73.58 | 5294 |

Table S4: Number of SNPs obtained in stacks by varying the level of missing data. The average level of missing data was calculated in PLINK 1.07 [7] (url: http://pngu.mgh.harvard.edu/purcell/plink/).
