# Supplementary figures and images for: Effect of diversity and missing data on genetic assignment with RAD-Seq markers
Source: BMC Res Notes. 2014 Nov 25;7:841. doi: 10.1186/1756-0500-7-841 (PMC4256836; doi:10.1186/1756-0500-7-841)

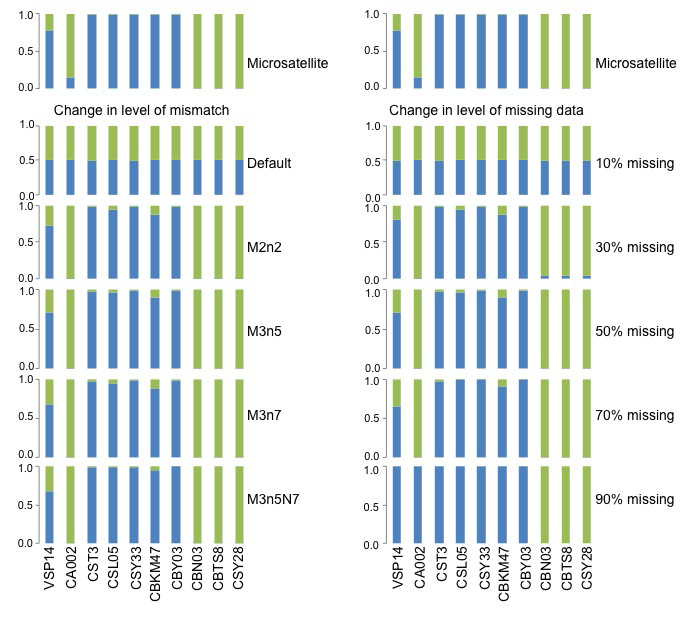

Supplement: Supplementary file 2 — Additional file 2: Figure S1: Bar plot of ancestry coefficient for individuals a) with increase in number of mismatches allowed to generate loci in Stacks and b) increase in proportion of missing data. (TIFF 554 KB) [file 13104_2014_3365_MOESM2_ESM.tiff]
